# A computational reconstruction of *Papio* phylogeny using *Alu* insertion polymorphisms

**DOI:** 10.1186/s13100-018-0118-3

**Published:** 2018-04-05

**Authors:** Vallmer E. Jordan, Jerilyn A. Walker, Thomas O. Beckstrom, Cody J. Steely, Cullen L. McDaniel, Corey P. St. Romain, The Baboon Genome Analysis Consortium, Kim C. Worley, Jane Phillips-Conroy, Clifford J. Jolly, Jeffrey Rogers, Miriam K. Konkel, Mark A. Batzer

**Affiliations:** 1 Department of Biological Sciences, Louisiana State University, 202 Life Sciences Building, Baton Rouge, LA 70803 USA; 2 Human Genome Sequencing Center, Baylor College of Medicine, Houston, TX 77030 USA; 3 Department of Molecular and Human Genetics, Baylor College of Medicine, Houston, TX 77030 USA; 4 Department of Neuroscience, Washington University School of Medicine, St. Louis, MO 63110 USA; 5 Department of Anthropology, New York University, New York, NY 10003 USA; 6 Department of Genetics & Biochemistry, Clemson University, Clemson, SC 29634 USA

**Keywords:** *Alu*, Retrotransposon, Phylogeny, Primates, Taxonomy, Evolutionary genetics, *Papio*, Hybridization

## Abstract

**Background:**

Since the completion of the human genome project, the diversity of genome sequencing data produced for non-human primates has increased exponentially. *Papio* baboons are well-established biological models for studying human biology and evolution. Despite substantial interest in the evolution of *Papio*, the systematics of these species has been widely debated, and the evolutionary history of *Papio* diversity is not fully understood. *Alu* elements are primate-specific transposable elements with a well-documented mutation/insertion mechanism and the capacity for resolving controversial phylogenetic relationships. In this study, we conducted a whole genome analysis of *Alu* insertion polymorphisms unique to the *Papio* lineage. To complete these analyses, we created a computational algorithm to identify novel *Alu* insertions in next-generation sequencing data.

**Results:**

We identified 187,379 *Alu* insertions present in the *Papio* lineage, yet absent from *M. mulatta* [Mmul8.0.1]. These elements were characterized using genomic data sequenced from a panel of twelve *Papio* baboons: two from each of the six extant *Papio* species. These data were used to construct a whole genome *Alu*-based phylogeny of *Papio* baboons. The resulting cladogram fully-resolved relationships within *Papio*.

**Conclusions:**

These data represent the most comprehensive *Alu-*based phylogenetic reconstruction reported to date. In addition, this study produces the first fully resolved *Alu-*based phylogeny of *Papio* baboons.

**Electronic supplementary material:**

The online version of this article (10.1186/s13100-018-0118-3) contains supplementary material, which is available to authorized users.

## Background

The burgeoning diversity and availability of whole genome sequencing (WGS) data offers intriguing possibilities for the field of comparative primate genomics. Currently, WGS data are publicly available for over 100 primate species (NCBI Resource Coordinators 2016). Traditionally, significant interest in the genetics of non-human primates stems from their sustained role as popular research models for studying human biology and evolution [1–5]. One such primate—well established as a model for human genetics and disease susceptibility—is the *Papio* baboon [6–11]. In addition to close genetic relatedness, the temporal and ecological landscape of early *Papio* evolution bears striking resemblance to that of early hominins [2, 12–14]. Both include ancient episodes of admixture, as well as migration out of Africa into the Arabian Peninsula during the Pleistocene [2, 15–20]. Appropriately, *Papio* baboons represent an intriguing model for human evolution.

*Papio* baboons occupy the largest geographical distribution of any non-human primate genus on the African continent [21–23]. These ground dwelling Old World monkeys inhabit most of sub-Saharan Africa, to the exclusion of the tropical rainforests of West Africa and the Congo Basin, and also extend into the south-western region of the Arabian Peninsula [24, 25]. *Papio* systematics have been extensively studied over the past 60 years with much debate as to which forms warrant species status [25]. The disagreement is in essence philosophical, centered on the question of what constitutes a species*.* However, recent studies employ a phylogenetic species concept [26–29], positing that consistent differences in physical appearance, ecology and social behavior justify the recognition of six extant species: *P. anubis*, *P. hamadryas*, *P. papio*, *P. cynocephalus*, *P. ursinus and P. kindae*. In this study, we recognize all six as species.

Despite considerable interest in *Papio* systematics, a fully resolved consensus phylogeny remains undetermined [20, 26, 30]. Interfertility has been documented between all neighboring species, with persisting natural hybrid zones in several regions where distinct morphotypes (species) come into contact [27, 31–36]. Thus, discordance between mitochondrial, morphological, and nuclear phylogenetic reconstructions could in part stem from a dense history of admixture and reticulation persisting throughout the course of *Papio* evolution. Mitochondrial based phylogenies support the divergence of *Papio* into northern and southern lineages [26, 30]. Individuals belonging to *P. anubis, P. papio* and *P. hamadryas* are consistently placed within the northern clade; with individuals belonging to *P. kindae* and *P. ursinus* comprising the southern clade. In these analyses, however, the placement of *P. cynocephalus* remains unclear with individuals found in both clades. In addition, such reconstructions have proven unsuccessful at resolving phylogenetic relationships within each clade. Thus additional analyses employing novel methodologies could further serve to elucidate evolutionary relationship within *Papio*.

*Alu* elements are well-established DNA markers for the study of systematic and population genetic relationships [37–47]. In part, they are effective evolutionary characters because of their high copy number in primate genomes and sustained mobilization throughout the course of primate evolution (~ 65 MY) [48–50]. Over 1.2 million copies have been identified in the human genome [51], with similar numbers reported for all other haplorrhine genomes sequenced to date [52–55]. *Alu* elements are discrete primate-specific DNA sequences (~ 300 bp) belonging to a class of non-LTR (long terminal repeat) retrotransposons termed short interspersed elements (SINEs). Following the transcription of a SINE, the mRNA sequence can be reverse transcribed into DNA, producing a new copy at a novel position in the host genome [56–58]. Over time, this process known as target primed reverse transcription (TPRT) can exponentially increase the retrotransposon content of a host genome. *Alu* elements, as well as all other SINEs, lack the requisite enzymatic machinery for TPRT; thus they require proteins encoded by larger retrotransposons known as LINEs (long interspersed elements) [48, 59, 60].

SINEs are valuable evolutionary characters because they can be assumed to be identical by descent, meaning that insertions shared between individuals were inherited from a common ancestor, rather than acquired by independent events [61]. Additionally, retrotransposons have known directionality [62, 63], with the ancestral state being the absence of the insertion. *Alu* elements are popular retrotransposon markers because their short length makes them particularly easy to assay using standard PCR. Considered nearly homoplasy-free [48, 49], most potential sources of homoplasy involving *Alu* elements can be resolved through Sanger sequencing [41, 42, 61]. Recent studies demonstrate the utility of *Alu* elements for *Papio* species identification, as well as retrieving population structure within distinct *Papio* species [28, 29]. Furthermore, *Alu* elements have been successfully used to resolve controversial relationships between primates [38, 39, 42, 64]. However, little is known about the efficacy of *Alu* elements to resolve phylogenetic relationships involving high levels of admixture.

Although a high-quality reference assembly currently exists for only one *Papio* species (*P. anubis*), WGS data have been generated for individuals representing all six *Papio* species through the Baboon Genome Consortium. Thus it is possible to conduct a comprehensive whole genome analysis of *Papio* phylogeny using *Alu* polymorphisms between species of the genus. For the present study, we created a computational pipeline to identify and characterize recently integrated *Alu* elements polymorphic within the genus *Papio*. These *Alu* insertion polymorphisms were used to reconstruct phylogenetic relationships within *Papio*. By utilizing *M. mulatta* as our reference, our approach placed equal evolutionary distance between each *Papio* diversity sample and the reference assembly [Mmul8.0.1]. The computational analyses performed in this study generated a well-supported phylogeny of *Papio* baboons and represents the most comprehensive *Alu*-based phylogenetic analysis reported to date. In addition, we report a novel approach to admixture and reticulation analysis using *Alu* insertions.

## Methods

### Samples

Whole-genome sequencing was performed by the Baylor College of Medicine Human Genome Sequencing Center on a panel of fifteen *Papio* baboons: four *P. anubis,* two *P. papio*, two *P. hamadryas,* three *P. kindae*, two *P. cynocephalus*, and two *P. ursinus*. In order to sample an equal number of individuals from each species, we used two individuals from each of the six extant *Papio* species (we randomly selected two individuals from *P. anubis* and *P. kindae*) to conduct our computational analysis. Lastly, our panel included WGS data from the macaque sample used to build the latest *M. mulatta* assembly [Mmul8.0.1] (Additional file 1)*.*

WGS data were accessed from the NCBI-SRA database [65]. The SRA-toolkit (fastq-dump utility) [66] was used to download paired-end next generation sequencing reads and convert them from .sra files to interleaved fastq files. We then used nesoni (https://github.com/Victorian-Bioinformatics-Consortium/nesoni; last accessed March 2018) to prune all known adapters, cleave bases with a phred quality score of 10 or lower, and exclude reads shorter than 24 base pairs in length. Two output fastq files were produced: one containing clean paired-end reads (both reads passed the nesoni filter), and a second containing unpaired orphan reads (one of the paired-end reads was excised).

### Polymorphic *Alu* insertion detection

**Fig. 1:**
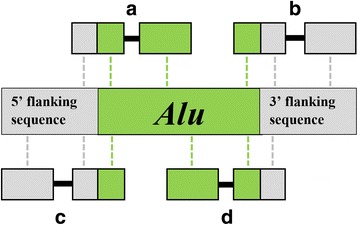
Computational detection of *Alu* insertion polymorphisms using split-reads. *Alu* insertions were identified using sequencing reads spanning the *Alu* integration locus whether these split-reads spanned the 5′ (**a** and **c**) or the 3′ (**b** and **d**) end of the insertion. The four split-reads represented in this figure are labeled **a**, **b**, **c**, and **d**. Green boxes represent *Alu* sequence; gray boxes denote flanking sequence. If the split-read is paired and its read-pair mapped to the flanking sequence (**b** and **c**), these mapping coordinates were used to provide additional support for the location predicted by the split-read. If the split-read’s read-pair mapped to the *Alu* (**a** and **d**), this was used to provide additional support for the presence of the predicted *Alu* insertion

We developed a computational pipeline to identify and characterize recently integrated *Alu* elements in paired-end next-generation sequencing (NGS) data. Our approach targeted young *Alu* insertions still polymorphic within the panel of individuals listed in the previous section. The approximate chromosomal position of each candidate insertion was estimated using a split-read method (Fig. 1). The resulting genotypes, generated for all individuals in our panel, revealed markers that provided phylogenetic signal.

The *Alu*Y subfamily has been identified as youngest and most active *Alu* subfamily in Simiiformes [48, 67–69]. Thus, in the alignment phase, we used BWA mem [70] to map paired-end NGS reads to a consensus *Alu*Y sequence obtained from Repbase [71]. Individual reads were required to map to either the head (5′) or tail (3′) of the *Alu*Y consensus sequence. In addition, reads mapping to the head of an *Alu* insertion were required to contain at least 15 bp of unmapped/non-*Alu* sequence directly upstream of the (5′) start of the *Alu* sequence. Likewise, reads mapping to the tail of the consensus *Alu* sequence were required to contain no less than 15 bases of unmapped sequence directly flanking the (3′) end of the sequence. Reads were mapped to the *Alu*Y consensus twice: once using the standard BWA mem parameters, and a second time using more liberal parameters (described in Additional file 2). Split-reads identified using standard parameters were later used to predict the location of an *Alu* integration site, while those identified during the liberal run were used simply to provide additional support for the insertion event. The *Alu* portion of each candidate split-read was then cleaved and remaining sequence aligned to Mmul8.0.1 using bowtie2 [72]. Split-reads were categorized as sequences that mapped uniquely to the *Alu*Y consensus and the Mmul8.0.1 assembly.

The approximate genomic position of each candidate insertion was calculated directly from the mapping positions of split-reads to Mmul8.0.1 and the *Alu*Y consensus. *Alu* insertion orientation was inferred from the alignment orientation of the supporting reads when mapped to the *Alu*Y consensus and Mmul8.0.1 assembly. During this phase the integration orientation of each candidate insertion was predicted in the forward orientation if positioned 5′ to 3′ on the sense strand, and the reverse orientation if positioned 5′ to 3′ on the anti-sense strand. If a split-read mapped in the same orientation to the consensus *Alu*Y and the Mmul8.0.1 assembly, it was predicted in the forward orientation. If the alignment orientations were discordant, the insertion was predicted in the reverse orientation.

Approximate genomic positions for non-reference (absent in Mmul8.0.1) *Alu* insertions, predicted in any of the 12 *Papio* individuals, were concatenated into a comprehensive list with the goal of identifying phylogenetically informative markers. All of these insertions were predicted from split-reads obtained during the standard *Alu* alignment run. In principle, phylogenetically informative *Alu* elements would have integrated into the *Papio* lineage following its divergence from *Macaca*. Thus, insertions shared between *Papio* and the *Macaca mulatta* sample were excluded. Likewise *Alu* elements identified in only one *Papio* sample were phylogenetically-uninformative, and thus were also excluded from this portion of the study. The remaining loci were genotyped in every individual on the panel. The three possible genotypes – homozygous present, homozygous absent, and heterozygous – were determined by analyzing sequences spanning the insertion locus. It was initially assumed that an individual was homozygous present for every insertion predicted in that sample. Likewise, it was initially assumed that an individual was homozygous absent for every locus not predicted in that individual. Insertions initially determined to be homozygous present were then re-evaluated to determine if they were in fact heterozygous present. Heterozygosity was determined by evaluating reads that mapped uniquely to the Mmul8.0.1 assembly. An insertion was reclassified as heterozygous if we identified reads in that individual that mapped continuously (without interruption) through the homologous empty site in the Mmul8.0.1 assembly. This empty site was defined as a sequence containing at least 15 bp of flanking both upstream and downstream from the predicted insertion locus. Additionally, if a homozygous absent genotype was predicted in a region with a local read-depth less than two standard deviations from the global mean, the genotype was instead considered unknown.

### PCR validation

The performance of the algorithm used in this study was assessed by comparing PCR validations performed for 494 loci in a panel of six *Papio* baboons: one from each extant *Papio* species [29]. From this dataset, our algorithm correctly predicted 98% of the PCR-validated events for presence/absence. In addition, the correct genotype (homozygous present, homozygous absent, or heterozygous) was computationally predicted for 93% of all events.

### Basal divergence analysis

Previous phylogenetic analyses support the ancestral divergence of *Papio* into two clades: northern and southern lineages [26, 30]. To evaluate this hypothesis we created a computational method to identify the basal divergence model best supported by our *Papio* dataset. A genus comprised of six species with three different possible phylogenetic topologies generates 31 different unique models for estimating the basal divergence (Additional file 3). For each model we determined the total number of insertions that supported and conflicted with each basal divergence. We calculated the standard deviation and z-score for each model. The model with the highest z-score represents the basal divergence model best supported by the dataset.

### Phylogenetic analysis

We used the model representing the basal divergence with the highest z-score (described in the previous section) as a pre-condition for our phylogenetic analysis. A comprehensive list of *Alu* insertions supporting this model (consistent with the north-south split hypothesis) were used to further resolve phylogenetic relationships within *Papio.* A heuristic search was performed using PAUP* 4.0b10 [73]. Since it is assumed that the absence of an *Alu* insertion is the ancestral state of each locus, Dollo’s law of irreversibility [74] was used in the analysis. Thirteen individuals were evaluated in this analysis: 12 *Papio* baboons, two representing each of the six extant *Papio* species, along with the *M. mulatta* sample used to build the Mmul8.0.1 assembly. Each individual received a score for each locus based on its computationally derived genotype. The presence of an insertion was scored as “1” for a filled site and “0” for an empty site; unknown genotypes were scored as “?”. Using PAUP we conducted a heuristic search using genotype data from *Alu* polymorphisms concordant with the north-south split with *M. mulatta* set as the outgroup. All loci were classified as individual insertions and set to Dollo.up for parsimony analysis as described previously [41]. Ten thousand bootstrap replicates were performed with the maximum tree space set to all possible trees.

We wrote a series of Python scripts to sort *Alu* insertions into clusters based on which baboons shared the insertion. This allowed us to determine the total number of *Alu* insertions shared between different sets/combinations of baboons. Each cluster contained *Alu* insertions shared among a distinct combination of baboons, yet absent from all other samples. For example, one cluster contained all *Alu* insertions shared between the *P. cynocephalus* samples and the *P. kindae* samples, yet absent from all remaining samples. Another cluster was comprised of *Alu* insertions shared between all six northern baboons, yet absent from all six southern baboons. Each cluster represents the total number of insertions shared uniquely between a particular “combination/set” of baboons. The resulting clusters were then analyzed to identify patterns of shared *Alu* polymorphisms. Using this script we quantified the total number of *Papio* indicative *Alu-*insertions, markers present in all six extant *Papio* species, yet absent from the *M. mulatta* sample. Clade indicative *Alu* polymorphisms were defined as insertions present in every species belonging to one clade, yet absent from all individuals in the other clade. In addition, we evaluated patterns of shared *Alu* polymorphism exhibited within each clade. In this analysis, we identified *Alu* polymorphisms exclusive to either the northern or southern clade, yet not present in all species within that clade. Lastly, we quantified the total number of species indicative *Alu* elements, defined as *Alu* polymorphisms present in both individuals belonging to a species, yet absent from all other *Papio* individuals in our panel.

## Results

### Polymorphic *Alu* identification

WGS data for multiple *Papio* baboons were generated through the Baboon Genome Analysis Consortium and made available on NCBI. From this dataset we selected a diversity panel consisting of 12 *Papio* baboons: two from each of the six extant species. We then used our computational pipeline to process these WGS samples, targeting *Alu* insertions present in multiple diversity samples, yet absent from the latest *M. mulatta* reference assembly [Mmul8.0.1]. In total, we identified 187,379 *Alu* insertions fitting this criterion.

### Basal divergence modeling

We evaluated 31 distinct basal divergence models (see Methods), to determine the one best supported by our computational genotype data (Additional file 3: Figure S1). The model with the largest z-score divided the *Papio* genus into two lineages: a northern clade containing *P. papio*, *P. anubis*, and *P. hamadryas*; and a southern clade consisting of *P. cynocephalus*, *P. ursinus*, and *P. kindae* (Additional file 3: Table S1). Of the 187,379 non-reference insertions (not present in Mmul8.0.1) reported in the previous section, 123,120 were concordant with this north-south basal divergence model (~ 66%) and 64,259 (~ 34%) were discordant.

### *Papio* phylogeny

**Fig. 2:**
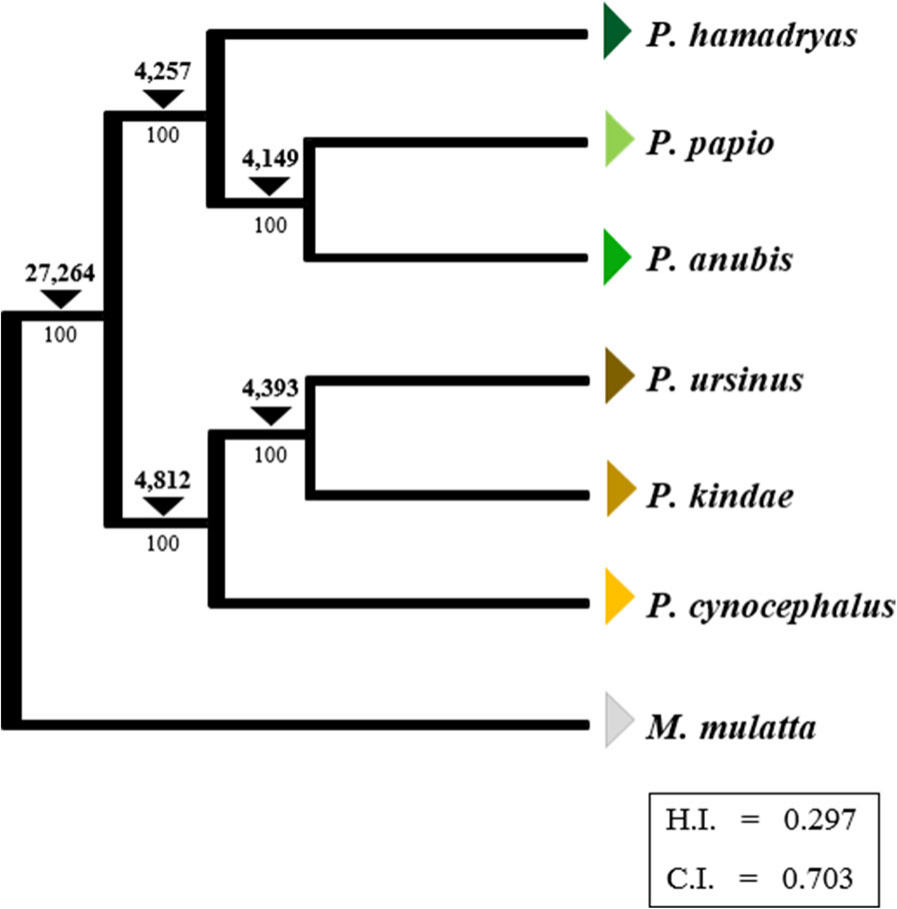
*Alu-*based phylogeny of extant *Papio* baboon species. Phylogenetic relationships of *Papio* baboons constructed using 123,120 *Alu* insertion polymorphisms. Genotypes computationally determined in 12 *Papio* baboons were used to construct a Dollo parsimony tree using *M. mulatta* as an outgroup. The percentage of bootstrap replicates (out of 10,000 iterations) is listed below each branch; the number of *Alu* insertions supporting each node is listed above each branch. Homoplasy index (H.I.) and consistency index (C.I.) are included below the cladogram

Using the data obtained from the panel of 12 *Papio* individuals, we constructed an *Alu-*based phylogeny of *Papio* baboons. For this analysis we used genotype data for 123,120 *Alu* insertions concordant with the north-south split hypothesis. The resulting cladogram resolved relationships within *Papio* with 100% bootstrap support at each node (C.I. = 0.703, H.I. = 0.297) (Fig. 2). Bootstrap values along with the total number of insertions supporting each node are included in Fig. 2.

**Fig. 3:**
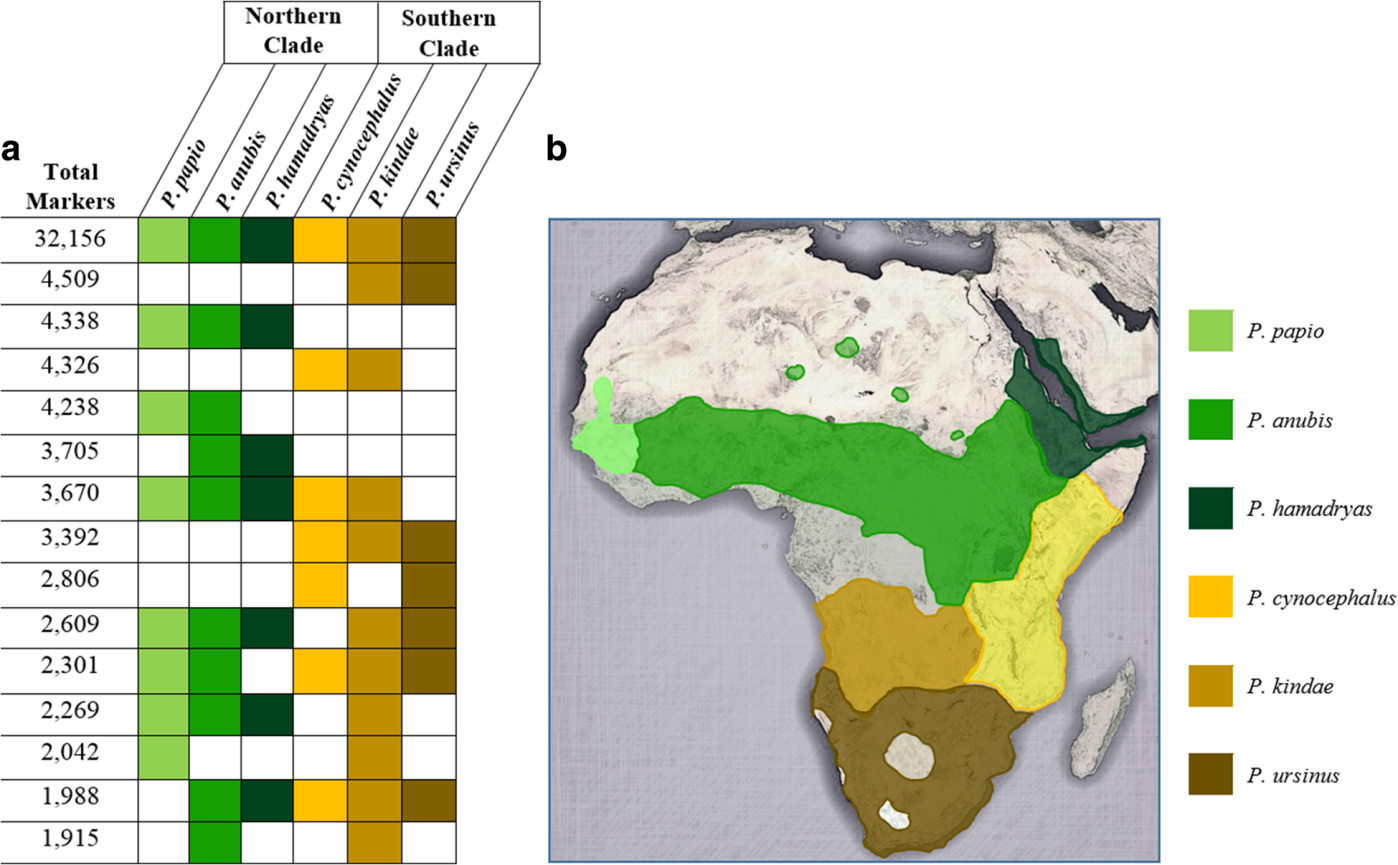
Common patterns of shared *Alu* insertion polymorphisms. **a** The number of *Alu* insertions shared exclusively between the species highlighted in each row. Markers were clustered based on precise presence/absence genotype data determined for six *Papio* baboons: one representing each *Papio* species. This figure displays the 15 largest clusters identified in this analysis. The colors correspond to the (**b**) Geographical distributions of the six *Papio* species. Map extrapolated from [30]. White/empty boxes indicate an empty site in that species

To further examine evolutionary relationships within *Papio, Alu* insertions shared among multiple samples were clustered according to the patterns of shared *Alu* insertion polymorphisms determined for our *Papio* samples. This analysis was conducted multiple times, using various combinations of individuals from each species. Regardless of the representative individual selected for each species, the rank and size of each cluster, remained consistent. However, because we were particularly interested in observing clusters formed between individuals belonging to different species, we used one representative sample from each species. In each species, we selected the individual with sequencing coverage closest to the average coverage determined across all samples (Additional file 1). The resulting clusters are displayed in Fig. 3. Of the 187,379 *Alu* insertions identified in all 12 samples, we retained only those shared among multiple individuals from our panel of six *Papio* individuals. In total, we identified 106,204 such elements grouped into 57 unique clusters (For the full table, see Additional file 4). Figure 3 displays the 15 largest clusters, representing a total of 76,264 *Alu* insertions (~ 72% of the dataset). The largest cluster contained 32,156 markers present in all six *Papio* species (Fig. 3). Seven of the eight next largest clusters were shared exclusively between baboons belonging to the same clade (north/south). In total, these seven clusters contained 27,314 *Alu* insertions (~ 26% of the dataset). Of the remaining clusters, four consisted of markers shared between five of the six *Papio* species (10,568 *Alu* insertions, ~ 10% of the dataset), and three clusters consisted of insertions shared between *P. kindae,* and at least one of the northern baboons (6226 *Alu* insertions, ~ 6% of the dataset).

**Fig. 4:**
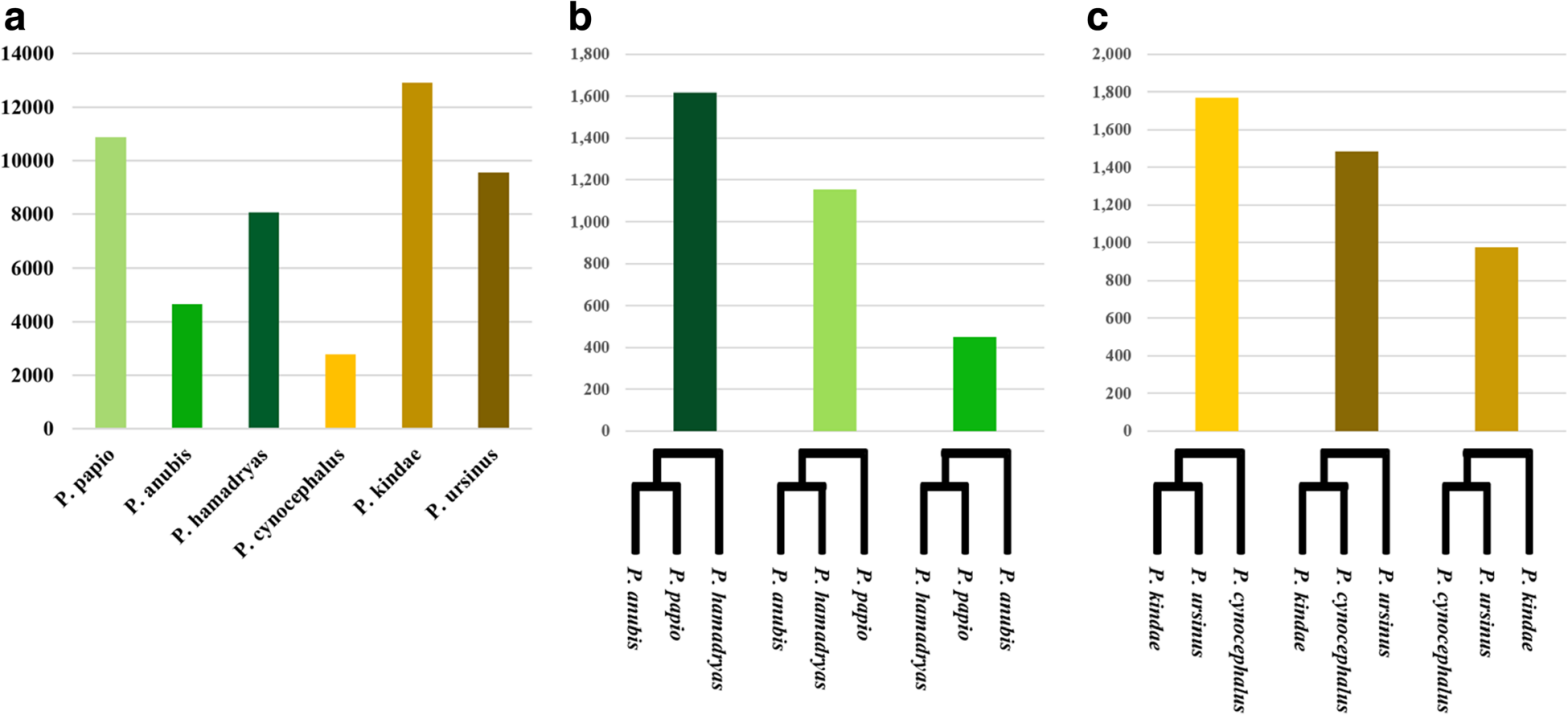
Analysis of phylogenetically informative *Alu* insertions. **a** Species indicative *Alu* insertion polymorphisms. For each species, the total number of *Alu* insertion polymorphisms shared exclusively between individuals belonging to that species. All species indicative markers were identified in multiple representative individuals. Also displayed is the number of *Alu* insertion polymorphisms supporting alternative northern (**b**) and southern (**c**) clade phylogenies. These markers were shared between multiple individuals belonging to each of the sister taxa displayed, yet absent from the third divergent species. Each phylogeny corresponds to the data point above it

Northern and southern clade phylogenies were then re-evaluated using all 12 *Papio* baboons: two from each of the six extant *Papio* species, with all 187,379 *Alu* insertions. *Alu* insertions shared exclusively between multiple individuals belonging to the same clade were classified as clade-specific markers. A total of 95,703 such markers were identified: 39,795 in the northern clade and 55,908 in the southern clade. These markers were clustered based on precise presence/absence genotypes determined for all 12 *Papio* baboons. Species indicative markers were defined as *Alu* insertions present in both individuals representing the same species, yet absent from all other members on the panel. In total we identified 48,808 species indicative markers: 23,578 markers were identified in the northern clade, with 25,230 identified in the southern clade. The total number of species indicative markers determined for each *Papio* species is displayed in Fig. 4a. Among northern baboon species, the highest number of species indicative *Alu* polymorphisms was determined for *P. papio* (10,873), followed by *P. hamadryas* (8060) and *P. anubis* (4645). In the southern clade, *P. kindae* reported the highest number of species indicative markers (12,891), followed by *P. ursinus* (9545)*,* and *P. cynocephalus* (2794). Furthermore we evaluated inter-species relationships by targeting clade-specific markers shared between all individuals belonging to two species within a clade, yet absent from both individuals from the remaining species. Within both clades, three unique clusters were formed from these data, each supporting a different clade phylogeny (Fig. 4b and c). A total of 7436 such elements was determined: 4220 in the northern clade and 3216 in the southern clade. Of the markers identified in the northern clade, 52% were shared exclusively between *P. anubis* and *P. papio* (1613 loci), 34% were shared between *P. anubis* and *P. hamadryas* (1153 loci), and the remaining 14% were shared between *P. papio* and *P. hamadryas* (450 loci). In the southern clade analysis, 43% of the *Alu* insertions were shared between *P. ursinus* and *P. kindae* (1766 loci), 36% were shared between *P. cynocephalus* and *P. ursinus* (1483 loci), and 28% were shared between *P. kindae* and *P. cynocephalus* (971 loci).

**Fig. 5:**
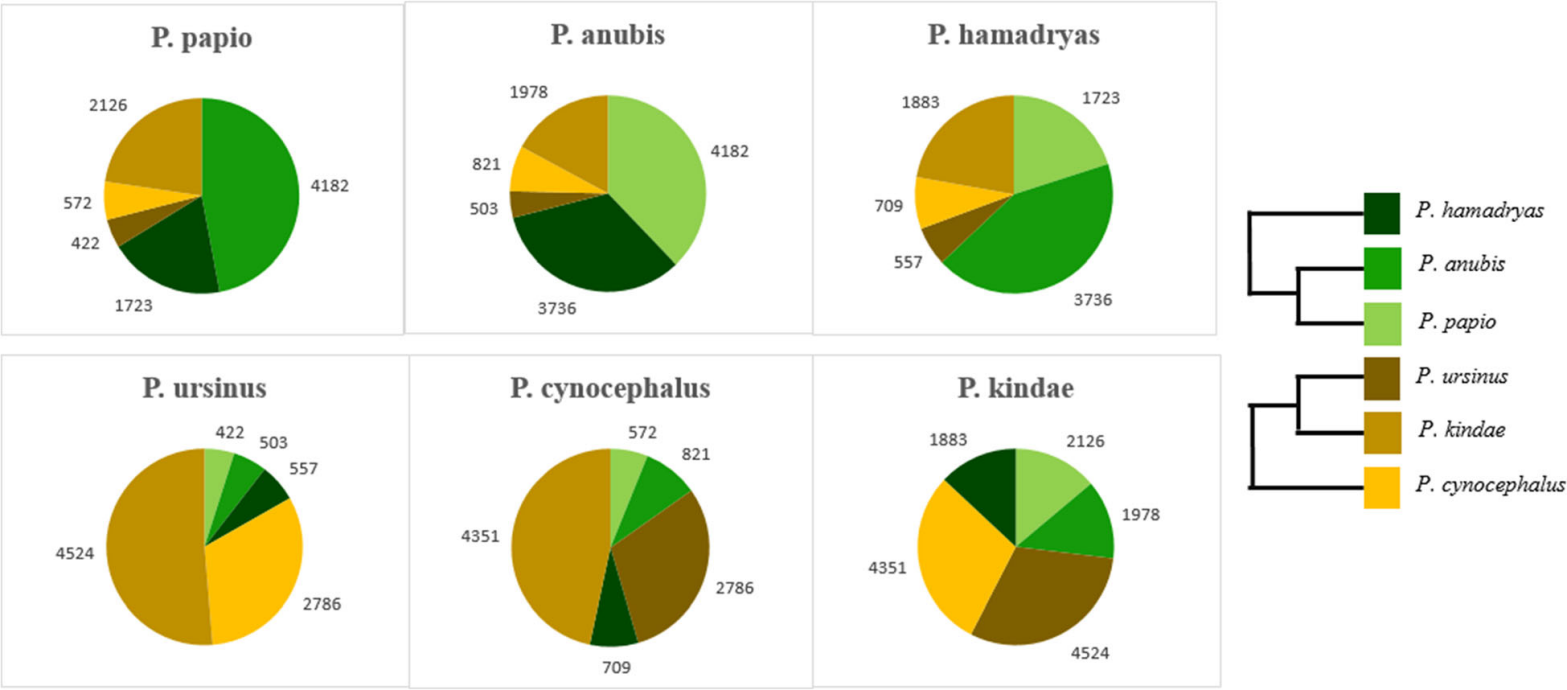
Low allele frequency *Alu* insertions polymorphic among *Papio* species. A diversity panel of 12 *Papio* baboons was used in this analysis: two representing each extant species. The only elements used in this analysis were those shared uniquely between two species. Each pie chart represents the average values determined from the two individuals representing that *Papio* species (the species name is listed above each pie chart). The size of every pie chart slice represents the number of *Alu* insertions shared between the species listed above that particular chart and the species represented by the color of the slice (indicated by the legend on the right). The numbers outside each pie chart correspond to the total number of *Alu* insertions represented by each slice

In addition, we evaluated low-allele frequency *Alu* polymorphisms using data obtained from our complete panel of 12 individuals: two representing each *Papio* species. *Alu* insertions used in this analysis were those shared uniquely between only two species, and absent from the other four. Thus the overall number of these insertions among *Papio* was relatively low. We clustered these elements based on their precise presence/absence genotypes. Clusters identified for each species are displayed in Fig. 5. The numbers of insertions listed corresponds to the average of the two individuals from each species. With the exception of *P. papio* and *P. hamadryas*, the largest clusters identified in *Papio* species contained *Alu* insertions shared between individuals belonging to the same clade (north/south). Although the single largest cluster identified in both *P. papio* and *P. hamadryas* consisted of elements shared with *P. anubis*, the second largest cluster was shared with *P. kindae.* All of the northern baboons shared more insertions with *P. kindae* than with the other two southern baboon species combined (*P. cynocephalus* and *P. ursinus*).

## Discussion

With the increasing availability of WGS data, admixture remains a fundamental challenge for evolutionary biologists. Nevertheless, the abundance of genomic data provides scientists the opportunity to use novel methodologies to re-examine complex evolutionary relationships. Well-documented extant hybrid zones coupled with a dense history of reticulation complicate the task of neatly organizing *Papio* baboons into a phylogenetic tree. Baboons are popular well-established research models for studying human disease and evolution, and therefore understanding the pattern of genetic variation within and between baboon species is important. As a result, an accurate and detailed understanding of *Papio* genomic evolution is quite valuable.

Despite the increasing availability of WGS data, high quality assemblies are not commonly constructed for multiple species belonging to the same genus. Instead, one individual is often used to build an assembly representative of an entire genus. However, often times WGS data are generated from individuals belonging to different species within that genus. For *Papio* baboons, a high quality (chromosome-level resolution) reference assembly exists only for *Papio anubis*, yet WGS data have been generated for multiple individuals from each extant *Papio* species. A traditional method used to identify *Alu* elements polymorphic within a genus involved identifying markers present in an assembly of interest, yet absent from the closest primate relative with a draft assembly. For *Papio* baboons, a lineage-specific *Alu* polymorphism would be defined as an element present in *P. anubis*, yet absent in rhesus macaques [as represented by the assembly Mmul8.0.1]. Since all of the subsequent markers would be identified in a *P. anubis* individual, this would introduce sampling bias towards markers present in *P. anubis*. However, our computational approach allowed us to align all of our representative *Papio* samples against the outgroup rhesus macaque [Mmul8.0.1], placing equal evolutionary distance between each *Papio* individual and the reference assembly. As a result, we were able to identify polymorphic *Alu* elements with minimal directional bias.

Analyses conducted using mitochondrial DNA support the most basal divergence of *Papio* into northern and southern clades. However, these analyses were unable to produce a phylogeny that fully resolved evolutionary relationships between *Papio* species. Our findings provide support for this basal north-south split hypothesis. Furthermore, this study produces the first whole genome computational analysis of *Alu* polymorphisms within *Papio*. By designing a computational method to detect and characterize *Alu* polymorphisms from multiple *Papio* individuals representing all known extant species and evaluating various basal divergence models, we were able to produce a fully resolved phylogeny of *Papio* baboons with 100% bootstrap support at each node.

In addition, our analysis of elements discordant with this phylogenetic model may offer insights into a complex history of admixture and reticulation within the *Papio* lineage. In the southern lineage, *P. kindae* shows the highest incidence of *Alu* insertions shared with the northern clade, yet absent from the other southern clade samples (11,286 elements). In total, we identified 64,259 elements discordant with topology of the phylogenetic tree (Fig. 2) that could be due to incomplete lineage sorting (ILS) or hybridization/admixture. Continued analyses involving a greater number of individuals would be necessary to accurately explain the taxonomic distribution of these insertions. Such analyses could potentially elucidate insertions indicative of speciation, the north-south split, hybridization, and many other evolutionary events. Thus, the data presented in this paper may be utilized to further evaluate *Papio* evolution. Such studies are likely necessary given the rich diversity that exists within the genus *Papio.* Furthermore, this approach has outstanding potential to inform analyses of other primate genera with complex evolutionary histories (e.g. *Cercopithecus, Macaca, Chlorocebus, Aotus, Microcebus, Saimiri* and others).

Contemporary arguments in favor of applying a phylogenetic species concept to the *Papio* genus rely heavily on the rich species diversity exhibited between morphotypes. Our findings provide support for the genetic diversity that exists within the genus *Papio.* In each extant species, we found an average of over 8000 elements shared exclusively between members belonging to that species. Despite previous debate as to whether *P. kindae* warrants species level classification, the largest number of species-specific elements characterized in this study were identified in *P. kindae* (12,891)*.*

One limitation of this study is that it is based on only 12 *Papio* individuals: two representing each species. It is very likely that the genetic diversity observed in each individual does not comprehensively represent diversity existing within the species as a whole. Each wild *Papio* species occupies a large range across the African continent; thus proximity to hybrid zones may contribute to interspecies diversity that is not captured in this analysis. Several species occupy ranges that contact other *Papio* species (Fig. 3b). Little is known about within species diversity. Only through further large-scale sampling and analyses can this be evaluated.

## Conclusions

In conclusion, this study exhibits the utility and efficacy of a whole genome analysis of *Alu* polymorphisms for resolving controversial phylogenetic relationships. In addition, it demonstrates the importance of employing diverse methodologies. Knowledge of the initial divergence of *Papio* into northern and southern clades, produced by previous studies and supported in this study, was instrumental in our analysis of *Papio* evolution. Despite high incidence of hybridization and sustained hybrid zones, we were able to produce a highly supported cladogram, resolving relationships within both the northern and southern clades. These data represent the most comprehensive *Alu-*based phylogenetic reconstruction reported to date. In addition, this study also produces the first fully resolved *Alu-*based phylogeny of *Papio* baboons. Our approach may offer useful applications for investigating other unresolved branches of the primate evolutionary tree.

## Additional files

Additional file 1: Sequencing information for the 13 WGS samples used in this study. Individuals listed in bold indicate the panel of *Papio* samples used to conduct the clustering analysis in which one representative sample was used for each species. All of the links provided in this file were last accessed March 2018. (XLSX 12 kb)

Additional file 2: An outline detailing the programs utilized in the computational pipeline. Command line arguments used in each run are provided. (DOCX 14 kb)

Additional file 3: An excel file containing a figure and table representing possible basal divergence model reconstructions generated using all six extant *Papio* species. A maximum of 31 rooted monophyletic models can be generated from such a genus comprised of six species. These models can be further organized into three distinct groups based on the number of species contained in the subsequent clades. Group I depicts the six different scenarios when one of the six species diverges prior to the other five. Group I-A) illustrates *P. kindae* diverging first, followed by B) *P. ursinus* first, then C-F) *P. cynocephalus, P. papio, P. hamadryas,* and *P. anubis* diverging first, respectively. Group II depicts the 15 different models when two of the six species diverge prior to the other four. All possible combinations of this scenario are illustrated in Group II A-O. Group III depicts the ten different models generated from a basal divergence that forms two clades each comprised of three species. All ten combinations are listed in Group III A-J. The values listed correspond to the 31 possible phylogenetic models displayed in Figure S1. For each model, the number of concordant insertions are provided in the third column; the number of discordant insertions can be found in the fourth column. The z-score determined for the number of discordant insertions is listed in the last column. The lowest z-score (indicating smallest proportion of discordant elements in group) is shown in bold font and indicates scenario III-A to be the most likely basal divergence model. (DOCX 61 kb)

Additional file 4: An extension of Fig. 3. It is an excel file containing the complete cluster list: all 57 clusters identified in this analysis. (XLSX 13 kb)

Additional file 5: This file lists the members of the Baboon Genome Analysis Consortium as well as their contact information. (DOCX 13 kb)

## Abbreviations

LINE: Long interspersed element
LTR: Long terminal repeat
SINE: Short interspersed element
TPRT: Target-primed reverse transcription
WGS: Whole genome sequencing
