# Supplementary material for: A computational reconstruction of Papio phylogeny using Alu insertion polymorphisms
Source: Mob DNA. 2018 Apr 5;9:13. doi: 10.1186/s13100-018-0118-3 (PMC5885306; doi:10.1186/s13100-018-0118-3)
Supplement: Supplementary file 2 — An outline detailing the programs utilized in the computational pipeline. Command line arguments used in each run are provided. (DOCX 14 kb) [file 13100_2018_118_MOESM2_ESM.docx]

**Command Line Arguments**

1. **Fastq-dump**
   1. fastq-dump --split-spot -Z [accession number] > your.fastq
2. **Nesoni**
   1. nesoni clip: clipped --gzip no interleaved: your.fastq
3. **BWA**
   1. Standard run:
      1. bwa mem –t 8 –C path2ref path2fsq > path2out.bam
   2. Liberal run:
      1. bwa mem -t 8 –p –C -k 10 –T 15 path2ref path2fsq > path2out.bam
4. **bowtie2**
   1. bowtie2 [options] –p 8 –very-sensitive -x < path2ref_index> -U path2fsq -S outpath

**BWA OPTIONS [default]:**

-t INT Number of threads [1]

-k INT Minimum seed length. Matches shorter than INT will be missed. The alignment speed is usually insensitive to this value unless it significantly deviates 20. [19]

-p Assume the first input query file is interleaved paired-end FASTA/Q. See the command description for details.

-T INT Don’t output alignment with score lower than INT. This option only affects output. [30]

-C Append append FASTA/Q comment to SAM output. This option can be used to transfer read meta information (e.g. barcode) to the SAM output. Note that the FASTA/Q comment (the string after a space in the header line) must conform the SAM spec (e.g. BC:Z:CGTAC). Malformated comments lead to incorrect SAM output.

**Bowtie2 OPTIONS:**

Main arguments

-x <bt2-idx> The basename of reference genome index. We used bowtie2 to index the reference genome.

-U <r> Path to input fastq.

-S <sam> Path to output SAM file

-D <int> Max <int> consecutive seed extension attempts

-R <int> Max <int> attempt to “re-seed” reads with repetitive seeds

-N <int> Max <int> allowed mismatches

-L <int> sets the length of the seed substrings

-i <int> the interval between seed substring

--very-sensitive option is the same as running with options: -D 20 -R 3 -N 0 -L 20 -i S,1,0.50.
