# Supplementary material for: A computational reconstruction of Papio phylogeny using Alu insertion polymorphisms
Source: Mob DNA. 2018 Apr 5;9:13. doi: 10.1186/s13100-018-0118-3 (PMC5885306; doi:10.1186/s13100-018-0118-3)
Supplement: Supplementary file 3 — An excel file containing a figure and table representing possible basal divergence model reconstructions generated using all six extant Papio species. A maximum of 31 rooted monophyletic models can be generated from such a genus comprised of six species. These models can be further organized into three distinct groups based on the number of species contained in the subsequent clades. Group I depicts the six different scenarios when one of the six species diverges prior to the other five. Group I-A) illustrates P. kindae diverging first, followed by B) P. ursinus first, then C-F) P. cynocephalus, P. papio, P. hamadryas, and P. anubis diverging first, respectively. Group II depicts the 15 different models when two of the six species diverge prior to the other four. All possible combinations of this scenario are illustrated in Group II A-O. Group III depicts the ten different models generated from a basal divergence that forms two clades each comprised of three species. All ten combinations are listed in Group III A-J. The values listed correspond to the 31 possible phylogenetic models displayed in Figure S1. For each model, the number of concordant insertions are provided in the third column; the number of discordant insertions can be found in the fourth column. The z-score determined for the number of discordant insertions is listed in the last column. The lowest z-score (indicating smallest proportion of discordant elements in group) is shown in bold font and indicates scenario III-A to be the most likely basal divergence model. (DOCX 61 kb) [file 13100_2018_118_MOESM3_ESM.docx]

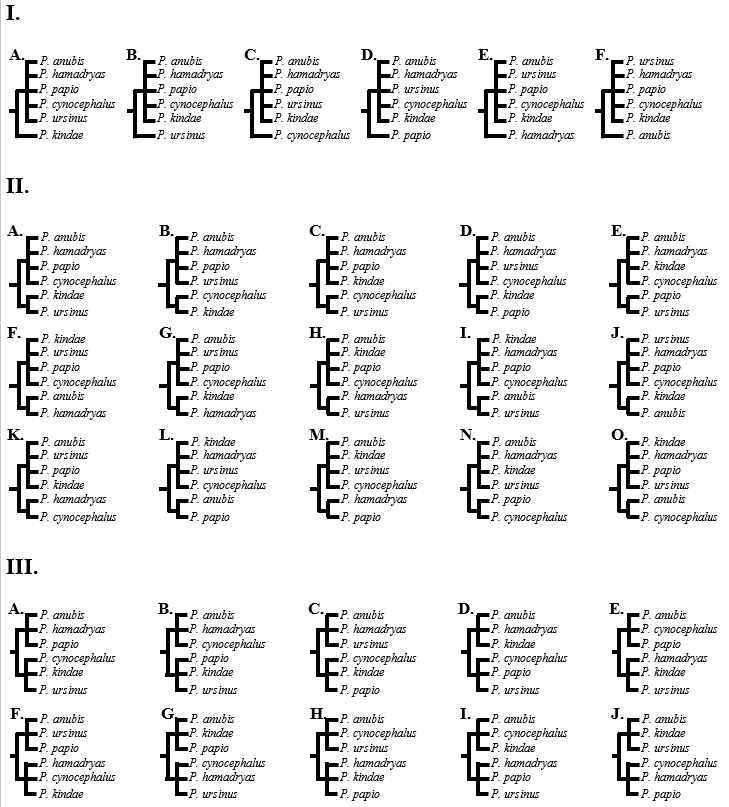


**Additional file 3: Figure S1 *Papio* basal divergence models**

Comprehensive representation of possible basal divergence model reconstructions generated using all six extant *Papio* species. A maximum of 31 rooted monophyletic models can be generated from such a genus comprised of six species. These models can be further organized into three distinct groups based on the number of species contained in the subsequent clades. Group I depicts the six different scenarios when one of the six species diverges prior to the other five. Group I-A) illustrates *P. kindae* diverging first, followed by B) *P. ursinus* first, then C-F) *P. cynocephalus, P. papio, P. hamadryas,* and *P. anubis* diverging first, respectively. Group II depicts the 15 different models when two of the six species diverge prior to the other four. All possible combinations of this scenario are illustrated in Group II A-O. Group III depicts the ten different models generated from a basal divergence that forms two clades each comprised of three species. All ten combinations are listed in Group III A-J.

**Additional file 3: Table S1 Statistical analysis of *Papio* basal divergence models**

|  |  | Concordant | Discordant | Z-score |
| --- | --- | --- | --- | --- |
| I | A | 56424 | 8523 | 0.292688 |
| I | B | 57072 | 7875 | -0.40374 |
| I | C | 58112 | 6835 | -1.52148 |
| I | D | 56821 | 8126 | -0.13398 |
| I | E | 56484 | 8463 | 0.228203 |
| I | F | 55265 | 9682 | 1.538313 |
|  |  |  |  |  |
| II | A | 55537 | 9410 | -1.30308 |
| II | B | 55004 | 9943 | -1.03091 |
| II | C | 56546 | 8401 | -1.81831 |
| II | D | 51607 | 13340 | 0.703724 |
| II | E | 51278 | 13669 | 0.871723 |
| II | F | 54501 | 10446 | -0.77406 |
| II | G | 52092 | 12855 | 0.456065 |
| II | H | 51674 | 13273 | 0.669511 |
| II | I | 50500 | 14447 | 1.268998 |
| II | J | 50820 | 14127 | 1.105595 |
| II | K | 52467 | 12480 | 0.264576 |
| II | L | 55389 | 9558 | -1.2275 |
| II | M | 53807 | 11140 | -0.41968 |
| II | N | 52160 | 12787 | 0.421342 |
| II | O | 51395 | 13552 | 0.811979 |
|  |  |  |  |  |
| III | A | 58017 | 6930 | **-2.72828** |
| III | B | 51544 | 13403 | 0.184783 |
| III | C | 50859 | 14088 | 0.493056 |
| III | D | 51660 | 13287 | 0.13258 |
| III | E | 52162 | 12785 | -0.09334 |
| III | F | 51388 | 13559 | 0.254988 |
| III | G | 52105 | 12842 | -0.06768 |
| III | H | 50922 | 14025 | 0.464704 |
| III | I | 50116 | 14831 | 0.82743 |
| III | J | 50773 | 14174 | 0.531759 |

The values listed correspond to the 31 possible phylogenetic models displayed in Additional file 3: Figure S1. For each model, the number of concordant insertions are provided in the third column; the number of discordant insertions can be found in the fourth column. The z-score determined for the number of discordant insertions is listed in the last column. The lowest z-score (indicating smallest proportion of discordant elements in group) is shown in bold font and indicates scenario III-A to be the most likely basal divergence model.
